# Can Damasio's Somatic Marker Hypothesis Explain More Than Its Originator Will Admit?

**DOI:** 10.3389/fpsyg.2020.607310

**Published:** 2021-01-15

**Authors:** Geir Overskeid

**Affiliations:** Department of Psychology, University of Oslo, Oslo, Norway

**Keywords:** emotion, problem solving, free will, determinism, choice

## Introduction

It is a very old conjecture that emotion governs human choice. Early philosophers like Epicurus (1993) and Aristippus of Cyrene [see Parry (2014)] appear to have held this assumption, often described as “psychological hedonism” [see Overskeid (2002)].

British thinkers of the 18th and 19th century were among the most important exponents of psychological hedonism. In a well-known dictum, Bentham (1823, p. 1) left little doubt as regards his own view: “Nature has placed mankind under the governance of two sovereign masters, pain and pleasure. … They govern us in all we do, in all we say, in all we think …”

Famously affirming that human rationality or reason could never on its own control behavior, Hume (1739) was equally clear. Indeed, he claimed (p. 413) that reason “can never oppose passion in the direction of the will.” Instead, said Hume (p. 415), “Reason is … the slave of the passions, and can never pretend to any other office than to serve and obey them.”

In the second half of the 20th century, psychologists and neuroscientists tended [with some exceptions, such as Toda (1980)] to stay away from the question of how reason and emotion interact to govern human conduct. Important in turning the tide, however, helping researchers see the role of emotions in thinking and decision making, were Damasio and his co-workers (e.g., Damasio, 1994; Bechara et al., 1997).

Damasio (1994) argued convincingly that there is no necessary conflict between reason and emotion—indeed, that emotion can support rational thought, and often does. To describe how cognition and emotion can interact when people choose, Damasio developed the somatic marker hypothesis (SMH). Central to the SMH is the assumption that people often do not choose on the basis of intellectual analysis alone, but also based on emotions elicited as part of the decision-making process. Verweij and Damasio (2019, p. 2) explain: “[T]he elements in this process—such as the options before us or the anticipation of the consequences of selecting one or another option—trigger emotive responses and generate the corresponding feelings or the corresponding covert signals, which can bias decisions nonconsciously.”

Though demonstrating that choice can be controlled by emotions, Damasio (1994) held that this is not always the case. He described several circumstances under which the somatic marker mechanism cannot, in his view, explain why people choose the way they do. On p. 173, he states, “Somatic markers may not be sufficient for normal human decision-making since a subsequent process of reasoning and final selection will still take place in many though not all instances.” He adds (p. 196) that “logical competence does come into play beyond somatic markers.”

Furthermore, says Damasio (1994, p. 177): “Some sublime human achievements come from rejecting what biology or culture propels individuals to do” —though “freedom from biological and cultural constraints can also be a hallmark of madness and can nourish the ideas and acts of the insane.”

These aspects of Damasio's hypothesis are worth discussing, since the SMH, though controversial, has been so influential—and the man behind the hypothesis has not changed his mind. A recent paper (Verweij and Damasio, 2019) repeats the claim that affect does not necessarily dictate our behavior. “On the basis of reasoning,” say Verweij and Damasio (2019, p. 3) “individuals are capable of making choices that are not in line with their emotive responses.”

## The Sublime

If we are to believe its originator, the SMH is unable to explain important aspects of the process behind human choice. What Damasio says about the SMH's limitations leaves open the possibility that another neurobiological mechanism will explain how reasoning determines choice. With regard to “sublime achievements,” this may not be the case, however. As we saw, some such deeds may result from the rejection of biological and cultural causation (Damasio, 1994, p. 177).

Darwin's thinking, for example, has been called sublime (e.g., Vucinich, 1989). It is easy enough to see that highly creative persons can reject beliefs that are prevalent in their culture. It is not so easy to understand what it means to reject what biology “propels individuals to do” (Damasio, 1994, p. 177). Was not the process of rejecting an idea or impulse an event in Darwin's nervous system—that is, in his biology? And furthermore, if people—even geniuses—are not propelled by their biology, what, then, is propelling them?

## Reasoning

There is also something in the reasoning process that can make a choice impervious to the effect of emotions, if we are to believe Damasio (1994). He does not specify how this may come about, though we saw him mention “logical competence.” He also states (p. 173) that after the somatic marker has done its job, there is “room for using a cost/benefit analysis and proper deductive competence …” A critic would note that a cost/benefit analysis and deductive competence can lead to new alternatives that a decision maker has to choose among. What is the mechanism that decides those choices? Damasio does not answer that question.

Damasio (2011) does claim, however, that emotions “are not the result of thinking through a problem and generating a solution.” This proposition would seem to conflict with evidence from several fields of research. There is considerable evidence that when problem solvers think through a problem and generate a solution, this can result in an emotion (e.g., Danek and Wiley, 2017). In a clinical domain, when cognitive therapy reduces or eliminates negative emotion, it appears to be because this helps patients think through a problem and generate a solution (e.g., DeRubeis et al., 2010).

The present author admires Damasio for his important work in many fields (e.g., Damasio and Maurer, 1978; Man and Damasio, 2019). The present author also admires David Hume, however—and Hume does not agree with Damasio. Indeed, Hume would favor a stronger SMH, without any exceptions, and he makes clear the reason why.

Says Hume (1739, p. 414), “. when we have the prospect of pain or pleasure from any object, we feel a consequent emotion of aversion or propensity, and are carry'd to avoid or embrace what will give us this uneasiness or satisfaction.” Hume cannot see why this should not always be the case. Making a choice is basically about simulating a possible future (however fleetingly) in which that choice has been made, and comparing it to at least one alternative possible future (e.g., Overskeid, 2000). This, then, is a situation in which our simulation has mapped “the prospect of pain or pleasure” that could result from choosing one or more alternatives. Hume (and others, as we have seen) assume that the emotion elicited in the process of choosing will determine the outcome of that process. Damasio, apparently, does not.

No author can answer every query a reader might have, and it is not incumbent upon Damasio to catalog all things that may affect a choice. Yet few questions in psychology have greater fundamental importance than those regarding how we choose. It may be reasonable, then, to ask why emotion can bias choice but not, in Damasio's view, determine it.

Furthermore, Verweij and Damasio (2019, p. 3) appear to say that affect does not necessarily dictate human choice, without referring to another mechanism that does. And Damasio (1994, p. 176–177) states, “[A]lthough biology and culture often determine our reasoning … and may seem to limit the exercise of individual freedom, we must recognize that humans do have *some* room for such freedom.” It may appear from these assertions that Damasio wants to avoid challenging the doctrine of free will. If that is the intention, Damasio should state clearly that in certain situations, a choice is exempt from causality—and ideally, he should explain when this is the case, and why.

Alternatively, Damasio may believe that when, “on the basis of reasoning” (Verweij and Damasio, 2019, p. 3), people make choices that are not determined by emotions, a different causal mechanism is at work. If this is the case, he should again say so—and if possible, describe the mechanism and characterize the situations in which this mechanism takes control of human choice (Though it's certainly true, as pointed out by a reviewer, that even if no such mechanism is suggested, this does not prove that none exists).

## How Much Can Damasio's Emotional Mechanism Explain?

Damasio [1994, see also Verweij and Damasio (2019)] claims, then, that when people engage in reasoning, their choices are not necessarily determined by an emotional mechanism. Of course, he could be right when he insists on the SMH's limitations—yet here are certain things we should consider.

Behind a choice that results from the ultimately biological process of reasoning, there must be a causal mechanism. There is much evidence that reasoning is governed by emotions, even when people end up choosing aversive options (Overskeid, 2000)—often because uncertainty feels even worse than distressing certitude (e.g., Gilbert, 2009).

Furthermore, people are cognitive misers [see Stanovich (2018)], and to start reasoning, we need to experience some difficulty in reaching a goal (in other words, a problem) [see Sanfey and Chang (2008)].

A goal is a state we want to reach. Not surprisingly, all generally accepted definitions of “problem” (e.g., Mayer, 1990) include a term such as “desire” or “want” —more specifically wanting to be in another state than the one we are currently in; a goal state, in other words.

Robinson et al. (2015) use “wanting” to refer to “visceral feeling of desire” (p. 107). Others speak of wanting in slightly different terms—but what the definitions have in common is that they describe emotion (e.g., Litt et al., 2010, Overskeid, 2000). It follows that *a problem is an emotional state*—it is a state of wanting. Reaching or not reaching a goal state are also typically described as events that elicit emotion [see Overskeid (2012)].

## Conclusion

Reasoning is a way of solving problems. But reason, says Hume (1739), is the slave of the passions. Supporting Hume, latter-day empirical studies indicate that reasoning is an inherently emotional process [see van Steenbergen et al. (2019)].

Damasio (1994), on the other hand, appears to claim that through processes like reasoning, insanity, and sublime achievement, one can evade the tyranny of emotions. Indeed, he says (p. 173–174): “The hypothesis does not concern the reasoning steps which follow the action of the somatic marker.” But perhaps the somatic marker keeps acting until the final choice is made. The way the present author sees it, Damasio has described a mechanism that changes Bentham's (1823) and Hume's (1739) propositions into more than mere conjectures. Emotions must necessarily decide all voluntary action—all the things we decide or choose to do. If Damasio does not agree, he should tell us why.

Some people oversell their insights. But might this be a story of underselling? Can the SMH explain more than its originator will admit?

## Author Contributions

GO confirmed being the sole contributor of this work and approved it for publication.

## Conflict of Interest

The author declares that the research was conducted in the absence of any commercial or financial relationships that could be construed as a potential conflict of interest.
